# Automatic gene annotation using GO terms from cellular component domain

**DOI:** 10.1186/s12911-018-0694-7

**Published:** 2018-12-07

**Authors:** Ruoyao Ding, Yingying Qu, Cathy H. Wu, K. Vijay-Shanker

**Affiliations:** 1 School of Information Science and Technology, Guangdong University of Foreign Studies, Guangzhou, China; 2 School of Business, Guangdong University of Foreign Studies, Guangzhou, China; 3 Department of Computer and Information Science, University of Delaware, Newark, DE 19716 USA

**Keywords:** Natural language processing, Gene ontology annotation, Relation extraction

## Abstract

**Background:**

The Gene Ontology (GO) is a resource that supplies information about gene product function using ontologies to represent biological knowledge. These ontologies cover three domains: Cellular Component (CC), Molecular Function (MF), and Biological Process (BP). GO annotation is a process which assigns gene functional information using GO terms to relevant genes in the literature. It is a common task among the Model Organism Database (MOD) groups. Manual GO annotation relies on human curators assigning gene functional information using GO terms by reading the biomedical literature. This process is very time-consuming and labor-intensive. As a result, many MODs can afford to curate only a fraction of relevant articles.

**Methods:**

GO terms from the CC domain can be essentially divided into two sub-hierarchies: subcellular location terms, and protein complex terms. We cast the task of gene annotation using GO terms from the CC domain as relation extraction between gene and other entities: (1) extract cases where a protein is found to be in a subcellular location, and (2) extract cases where a protein is a subunit of a protein complex. For each relation extraction task, we use an approach based on triggers and syntactic dependencies to extract the desired relations among entities.

**Results:**

We tested our approach on the BC4GO test set, a publicly available corpus for GO annotation. Our approach obtains a F1-score of 71%, a precision of 91% and a recall of 58% for predicting GO terms from CC Domain for given genes.

**Conclusions:**

We have described a novel approach of treating gene annotation with GO terms from CC domain as two relation extraction subtasks. Evaluation results show that our approach achieves a F1-score of 71% for predicting GO terms for given genes. Thereby our approach can be used to accelerate the process of GO annotation for the bio-annotators.

## Background

The Gene Ontology (GO) [1] is a resource that supplies information about gene product function using ontologies to represent biological knowledge. These ontologies cover three domains: (i) Cellular Component (CC), the parts of a cell or its extracellular environment; (ii) Molecular Function (MF), the elemental activities of a gene product at the molecular level, such as binding or catalysis; and (iii) Biological Process (BP), operations or sets of molecular events with a defined beginning and end, pertinent to the functioning of integrated living units (cells, tissues, organs, and organisms).

GO annotation is a process which assigns gene functional information using GO terms to relevant genes in the literature. It is a common task among the Model Organism Database (MOD) groups. Manual GO annotation relies on human curators assigning gene functional information using GO terms by reading the biomedical literature. Currently manual annotations are made by experienced biocurators from annotation projects including TAIR (http://www.arabidopsis.org/), Saccharomyces Genome Database (http://www.yeastgenome.org/), Mouse Genome Informatics (http://www.informatics.jax.org/), WormBase (http://www.wormbase.org/), PomBase (http://www.pombase.org/), FlyBase (http://flybase.org/), and ZFIN (http://zfin.org/). This process is very time-consuming and labor-intensive. As a result, many MODs can only afford to curate a fraction of relevant articles. For example, the curation team of TAIR has been able to curate less than 30% of newly published articles that contain information about Arabidopsis genes [2].

Many systems have been developed for automatic GO annotation. Zhu et al. [3] trained a logistic regression model to detect GO evidence sentences and developed a search-based approach to predict GO terms based on the GO evidence sentences. Li et al. [4] built a binary classifier to identify evidence sentences and developed an information retrieval-based method to retrieve the GO term which is most relevant to each evidence sentence. This method was based on a ranking function that combined cosine similarity and the frequency of GO terms in documents, and also used a filtering method based on high-level GO classes. Gaudan et al. [5] introduced a method for automatic identification of GO terms in natural language text. Their work was based on considering the proximity between the words occurring in text and the information content of the GO terms. Couto et al. [6] presented a system called GOAnnotator that automatically identifies evidence text in literature for GO annotation of UniProt/Swiss-Prot proteins. Emadzadeh et al. [7] proposed an approach for finding GO terms for different genes in an article. Their approach was based on distributional semantic similarity over the GO terms. Tuan et al. [8] introduced an approach using the GO cross products as the GO term representation to recognize GO terms in text. Chen et al. [9] proposed a rule-based GO evidence sentence retrieval systems based on a set of rule patterns.

Despite the fact that there are three types of GO terms (CC, MF, and BP) and the kind of textual evidences for each type are different, current approaches for automatic GO annotation tend to use a single approach for annotations of all types of GO terms. This may be the reason that previous methods are not good enough to be used in automatic GO annotation. In this paper, we present a novel approach of annotating genes using GO terms from the CC domain, where we cast the task of GO annotation as relation extraction between gene and other entities. Evaluation shows our approach achieves a F1-score of 71% (91% precision), thereby can be used by the bio-annotators to accelerate the process of GO annotation.

## Methods

Based on our study, we found that GO terms from the CC domain can be divided into two sub-hierarchies: (1) subcellular location terms: i.e., terms in GO under CC category, which are in the sub-hierarchy rooted by 19 GO terms (shown in Table 1, all of which have “cellular component” GO:0005575 as parent node). (2) protein complex terms: i.e., terms in GO under CC category, which are in the sub-hierarchy rooted by protein complex (GO:0043234). We treat the task of gene annotation using GO terms from these two sub-hierarchies as two relation extraction tasks: (1) extract cases where a protein is found to be in a subcellular location, and (2) extract cases where a protein is a subunit of a protein complex.

**Table 1:** 19 root GO Terms for Subcellular Location

Name	ID
*Extracellular region*	*GO:0005576*
*Cell*	*GO:0005623*
*Nucleoid*	*GO:0009295*
*Membrane*	*GO:0016020*
*Virion*	*GO:0019012*
*Cell junction*	*GO:0030054*
*Extracellular matrix*	*GO:0031012*
*Membrane-enclosed lumen*	*GO:0031974*
*Viral occlusion body*	*GO:0039679*
*Organelle*	*GO:0043226*
*Extracellular matrix component*	*GO:0044420*
*Extracellular region part*	*GO:0044421*
*Organelle part*	*GO:0044422*
*Virion part*	*GO:0044423*
*Membrane part*	*GO:0044425*
*Synapse part*	*GO:0044456*
*Cell part*	*GO:0044464*
*Synapse*	*GO:0045202*
*Symplast*	*GO:0055044*

### Relation extraction based on triggers and syntactic dependencies

We use trigger-based approach for the relation extraction tasks in this paper. A trigger will be a word or multi-word expression that indicates a relation. For example, in sentence “CSC-1 is a subunit of the Aurora B kinase complex” (**example sentence 1**), the relation between the protein complex and its subunit is indicated by word “subunit”. Similarly, the word “dimerizes” can be taken as a trigger for the relation between two protein complex components as indicated in the sentence “Mad1 dimerizes with Max” (**example sentence 2**).

**Fig. 1:**
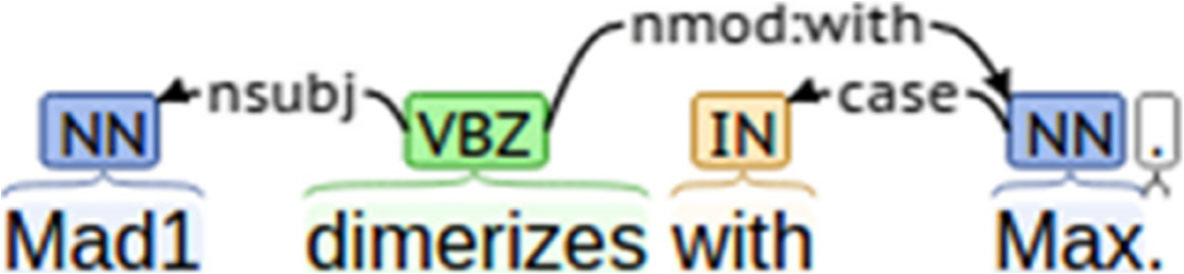
Syntactic dependencies for example sentence 2

Parsing the text where the trigger and the entities are mentioned can identify any syntactic dependencies between them. In our relation extraction approach, after detecting the triggers, we use rules based on the dependency edges between the trigger and the entities to see whether the entities fit into the arguments of the trigger to confirm the relation. For the extraction of the component-component relation from **example sentence 2**, consider Fig. 1 that shows the syntactic dependencies between the trigger and the two entities that are related.

To confirm the relation between “Mad1” and “Max”, our rule will enforce the following constraints.: (1) the trigger in a verb form appears in the sentence; (2) One of the entities, of type protein (“Mad1”), is the nominal subunit of trigger “dimerizes”; and (3) “Max” is the modifier of trigger “dimerizes”, where the modification relation is given by nmod: with.

**Fig. 2:**
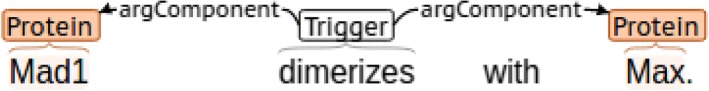
Approach output for example sentence 2

We use an existing framework in our lab to develop rules for confirming relations. Given one sentence, the framework uses the BLLIP parser [10, 11] to obtain the parse trees and then apples Stanford Conversion tool to get the Standard Dependency Graph. This framework provides a template to write rules that are in the form of a set of conditions and actions. Back to **example sentence 2**, after specifying the triggers and the syntactic dependency conditions, an “argComponent” edge is added from the trigger to each protein entity, as shown in Fig. 2.

**Fig. 3:**
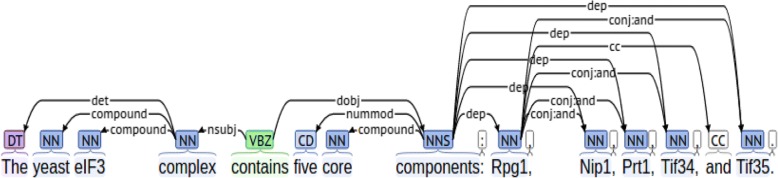
Syntactic dependencies for example sentence 3

**Fig. 4:**
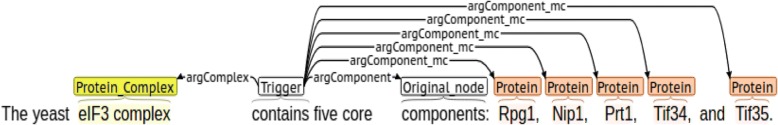
Approach output for example sentence 3

This framework also detects other extra-syntactic information such as is-a and member-collection relations and use them to propagate the original relations to form new relations. For example, Fig. 3 shows the syntactic dependencies constructed based on the BLLIP parser output for sentence “The yeast eIF3 complex contains five core components: Rpg1, Nip1, Prt1, Tif34, and Tif35.” (**example sentence 3**). Based on the syntactic dependencies, we can see the subject and object attached with “contains” are nodes “complex” and “components”. We just need to write rule to detect the relation between these two nodes. The framework will handle the member-collection structure and add new edges, as shown in Fig. 4.

### Gene annotation concerned with subcellular location

**Fig. 5:**
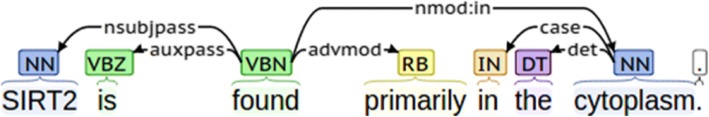
Syntactic dependencies for example sentence 4

This annotation for a protein requires the identification of text that relates a protein with a subcellular location. The first relation we are interested in is the *found-in* relation, where triggers such as “found” and “detected” are used. A key aspect of the rules for these triggers is the presence of a locative preposition such as “in”, “at” and “on”. Figure 5 shows the syntactic dependencies of the sentence “SIRT2 is found primarily in the cytoplasm.” (**example sentence 4**). Given the word “found” as trigger, we can see “SIRT2” is the nominal subject of the trigger, and “cytoplasm” is the modifier of the trigger (there is an “nmod:in” edge from “found” to “cytoplasm”). Thus, we can confirm the found-in relation between “SIRT2” and “cytoplasm”.

**Fig. 6:**
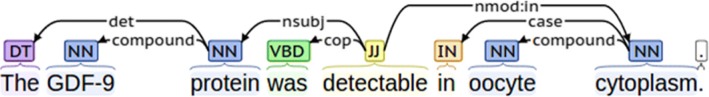
Syntactic dependencies for example sentence 5

Note the triggers do not need to appear only in the verbal forms but can also appear in their nominal forms. For example, the trigger “detected” is used in its nominal form “detection” as in the phrase: “the detection of ZmHK1 in endoplasmic reticulum”. The use of the trigger in adjectival form is illustrated in Fig. 6 (**example sentence 5**). Because of the use of the copular structure, the syntactic argument structure is similar to the verb case where the nsubj and nmod:in (or other locative preposition) identifies the protein and the location.

**Fig. 7:**
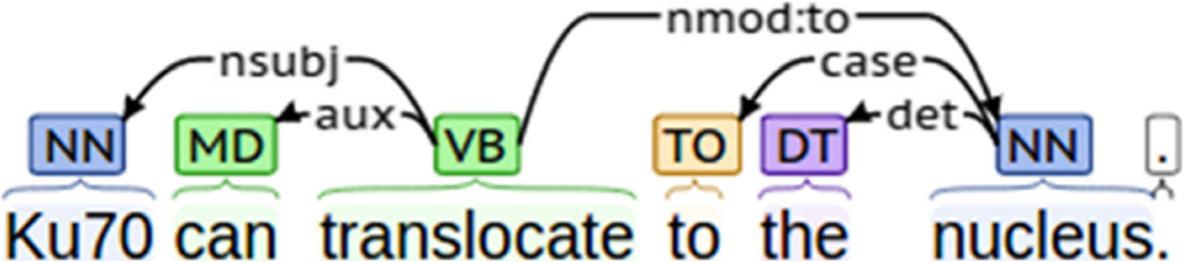
Syntactic dependencies for example sentence 6

In addition to the trigger words that correspond to a broadly-defined *found-in* relation, we also consider “*movement*” verbs. For these cases, we need to ensure that the moved entity is a protein and the new/old location is indicated by a locative prepositional phrase. An example of this kind can be found in Fig. 7 (**example sentence 6**). Unlike previous cases, since the verbs used as triggers indicate movement of their objects, these prepositions are likely to be “to” and “from”, rather than “in”, “on”, “at”.

**Fig. 8:**
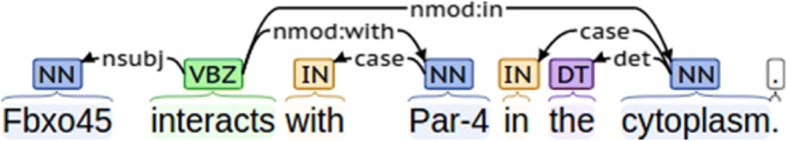
Syntactic dependencies for example sentence 7

The next type of relation we consider does not include a trigger that indicate the *found-in* or *movement* relation explicitly. Instead, this type of relation covers cases where the proteins are arguments of an event which occurred in some subcellular location and hence the protein-subcellular location relation is implied. For example, from sentence “Fbxo45 interacts with Par-4 in the cytoplasm” (**example sentence 7**), we can infer that “Fbxo45” and “Par-4” must both be in the cytoplasm since the interaction took place in this region. Figure 8 shows the syntactic dependencies of this sentence. From the graph, we can identify the proteins by considering the verb’s syntactic arguments such as “nsubj”, “nmod:with” and “dobj”. The nmod:in edge from the same verb to the location phrase indicates where the event took place.

**Fig. 9:**
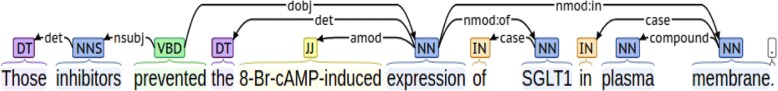
Syntactic dependencies for example sentence 8

Note that there are a variety of verb structures that can be used including passives. Also, the event need not be indicated by words in verbal forms but can also appear in their nominal forms. For example, in Fig. 9 (**example sentence 8**), we can know “SGLT1” must be in “plasma membrane” since it is expressed there.

If we were only interested in protein-subcellular location relation, then we can consider triggers that are adjectives that are modifiers specifying subcellular locations (e.g., nuclear and cytoplasmic). In such cases, we will look for the protein which these adjectives modify, e.g., in the phrase “the nuclear transcription factors AP-1 or NFIL-2A”. However, our interest here is not just in detection textual mentions of a protein’s subcellular location. Rather, we are interested in annotation of a protein. Thus, the text must indicate not only the relation but that it is a finding. Therefore, we will not consider cases such as “cytoplasmic CD3” in this work, because we believe it refers to existing knowledge.

In addition to the above rules, we also attempted to capture descriptions of experimental results on protein localization, through fluorescent microscopy. Consider the following text: “The localization of CeCDC-14 was analyzed in wild-type *C. elegans* embryos, using the affinity-purified anti-CeCDC-14 antibodies for immunofluorescence microscopy. Later in mitosis, during telophase, this staining compacted to a single dot that was positioned between the two daughter cells, highly reminiscent of the midbody”. The word “staining” above indicates protein localization. While we haven’t developed concrete rules for these cases, we look for location terms in the same sentence. In this case, we thus obtain the location of “midbody”. Next, we attempt to find which protein’s staining is detected or observed. For this purpose, we try to see if there is a unique protein (other than tags such as GFP) mentioned in the same or previous sentence. In this case, there is only one protein mentioned, i.e., “CeCDC-14”. We use this (as a low confident clue) to infer that protein “CeCDC-14” is in location “midbody”.

Finally, as for the detection of subcellular location mentions, we use a dictionary-based method, where the subcellular location dictionary is created using terms from GO subcellular location sub-hierarchies (described in Table 1). As for the detection of protein mentions, we use our existing gene normalization system, pGenN [12]. In this way, after we detect the relation between a protein and a subcellular location, the protein can be directly associated with a GO term.

### Gene annotation concerned with protein complex

In order to annotate a protein with a protein complex GO term, we will first detect the text where a protein is mentioned as a component of a protein complex. This can be done through detecting: (1) protein complex and component relation, and (2) protein complex component and component relation. After the relations are detected, we will infer the proper GO terms under the protein complex sub-hierarchy based on the text to annotate the protein.

We found the protein complex and component relation is often indicated by the “part whole” semantic structure, where a protein mention is found in the “part” argument position and a protein complex is found in the “whole” argument position. Consider sentence “CSC-1 is a subunit of the Aurora B kinase complex” (**example sentence 9**). In this case, the “part whole” structure is indicated by trigger “subunit”. However, the “part whole” structure can be indicated by more general English words. E.g., in sentence “MRE complex contains Sp1 or related proteins” (**example sentence 10**), the “part whole” structure is indicated by word “contains”.

We use three types of rules to extract the *part of* relation between a protein complex and its component from individual sentences. The first type of rules covers cases like “protein is a subunit of protein complex”, where the triggers are word such as “subunit”, and “component”. In this type of rules, the agent should be the protein, and the argument should be the protein complex. The second type of rules covers cases like “protein complex contains protein”, where the triggers are words such as “contains” and “consists”. In this type of rules, the agent should be the protein complex, and the argument should be the protein. The last type of rules covers cases like “protein is detected in protein complex”, where the triggers can be “detected-in” and “observed-in”. In this type of rules, the agent should be the protein, and the argument should be the protein complex.

**Fig. 10:**
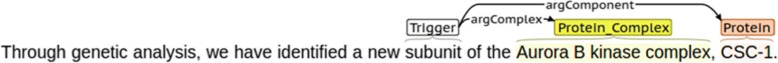
Approach output using type 1 rules for protein complex and component relation extraction

**Fig. 11:**
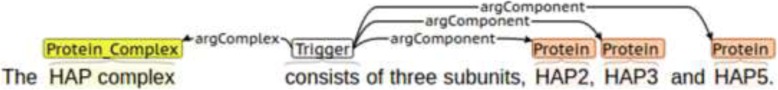
Approach output using type 2 rules for protein complex and component relation extraction

**Fig. 12:**
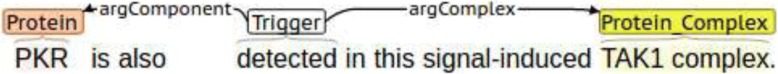
Approach output using type 3 rules for protein complex and component relation extraction

By specifying the triggers and the conditions, we add an “argComponent” edge from the trigger to the protein entity, and an “argComplex” edge from the trigger to the protein complex entity if the conditions are met. Figures 10, 11 and 12 show examples (**example sentence 11–13**) of the output using the three types of rules described in the previous paragraph.

**Fig. 13:**
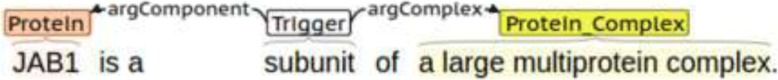
Approach output for example sentence 14

Noted that we only need to confirm whether a protein is mentioned as a component of a protein complex. To achieve this goal, for the protein complex and component relation extraction task in this paper, we do not need to limit ourselves to cases when the protein complex is named. Some general phrases that denote a protein complex such as “a protein complex” can also be used as valid context. For example, in Fig. 13 (**example sentence 14**), if we know “JAB1” is a subunit of a protein complex, this is enough for us to select this sentence as GO annotation evidence sentence. Thus, in addition to detecting protein complex names using a dictionary-based approach, where the dictionary is created based on PRO [13], Complex portal [14] and CORUM [15], we also detect phrases that denote a protein complex based on f-term. The notion of f-term was introduced in [16] and further developed in [17]. An f-term comes from a small list of words which can indicate the type of a named entity, such as protein complex. The regular expression combined with the protein complex f-terms we generated for identifying phrases that denote a protein complex is as follows:

“/(complex|dimer|trimer|tramer|hexamer|nonamer|tamer|decamer|octomer|oligomer)$/”.

To extract the protein complex component and component relation from individual sentences, we use two types of rules: (1) “protein and protein form a complex”, (2) rules based on protein protein interaction (PPI). For the latter, we first use a set of triggers and rules described in [18] to detect PPI relations. Then we use a heuristic that detects binding forms a complex (i.e., the interacting partners are in a component-component relation) if the text indicates that the PPI: (a) is stable, (b) performs some function, or (c) has more than two proteins involved. By specifying the triggers and the conditions, we add “argComponent” edges from the trigger to the protein entities if these conditions are met.

**Fig. 14:**
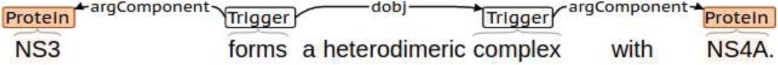
Approach output for example sentence 15

**Fig. 15:**
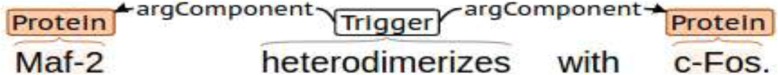
Approach output for example sentence 16

The first type of rules covers the most basic and straightforward cases. Triggers used here are “form” with any protein complex f-term such as “complex” and “trimer”. Noted that the word “form” and protein complex f-term do not need to be adjacent, as shown in Fig. 14 (in this example, i.e., **example sentence 15**, trigger f-term needs to be the object of trigger “form”). The first type of rules also covers cases like “protein complexes with protein”, as shown in Fig. 15 (**example sentence 16**), and the nominal form of the verb where words such as formation (of complex) and dimerization are used as triggers.

**Fig. 16:**
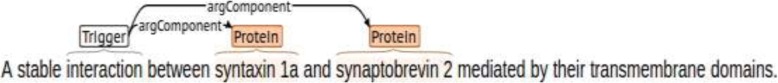
Approach output for example sentence 17

The second type of rules to extract protein complex component and component relation is based on the detection of the PPI relation. Triggers used for detecting the PPI relation are words such as “interact”, “bind”, and “associate”. We consider three cases where the PPI indicates protein complex component and component relation. Since a protein complex is often defined as a stable set of interacting proteins, the first case we consider is when the PPI is stable (PPI + stable), as shown in Fig. 16 (**example sentence 17**).

**Fig. 17:**
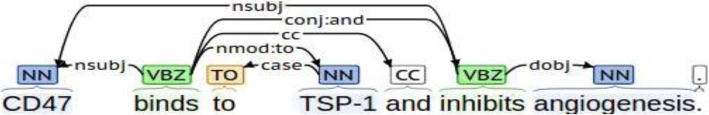
Approach output for example sentence 18

The second case we consider is when protein interacts with other protein to perform some function, or the interaction itself performs some function (PPI + function). Our hypothesis is that if the interaction results in some function being performed, it is stable enough or lasts long enough to be called a complex. For example, in Fig. 17 (**example sentence 18**), based on the trigger “binds” and the syntactic dependencies between the trigger and the two proteins “CD47” and “TSP-1”, we can confirm there is a PPI relation. Since there is a conjunction edge from the PPI trigger “binds” to another verb “inhibits”, we can infer that “CD47” binds to “TSP-1” to perform some function, i.e., “inhibits angiogenesis”.

**Fig. 18:**
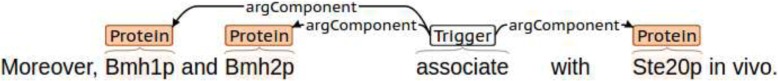
Approach output for example sentence 19

Finally, if more than two proteins are found to be involved in one interaction, we treat these proteins as components of one same protein complex (PPI + multiple components). Consider this sentence “Moreover, Bmh1p and Bmh2p associate with Ste20p in vivo” (**example sentence 19**). Since there are more than two proteins involved in the interaction, we can infer that those proteins are components of one same protein complex, as shown in Fig. 18.

**Fig. 19:**
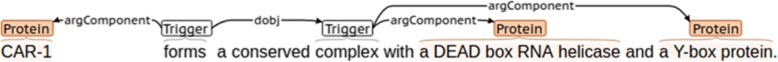
Approach output for example sentence 20

Noted that we do not need to require all the components involved to be explicitly named proteins. Thus, for example, the following sentence (Fig. 19, **example sentence 20**) suffices to assert that CAR-1 belongs to a complex.

After the relations are extracted, we will infer the proper GO terms under the protein complex sub-hierarchy based on the extracted information to annotate the protein. Since entries from PRO, Complex portal and CORUM can all be linked to GO entries, if the protein complex we recognized can be matched with the dictionary created from these resources, we can directly associate it with the corresponding GO terms. Note that we do not need to consider the species information during the dictionary lookup process, since GO terms are not species specific.

If we cannot figure out more information for the protein complex mentioned in the article, we will just annotate the proteins involved in the extracted relations with GO term “protein complex” (GO:0043234).

## Results

The BC4GO corpus [19] is a publicly available corpus for the GO annotation task. It consists of a set of articles and associates GO annotations for these articles. This corpus contains 200 full-text articles, 100 of them were designated for training, 50 for development, and the remaining 50 were used for testing. Annotations in this corpus include the PMID, Gene ID and GOID triplets (a list of relevant GO terms for genes in a paper).

From the BC4GO test set, we extract the PMID, Gene ID and GOID triplets with annotated GO terms from the CC domain. Altogether. 97 PMID, Gene ID, and GOID triplets are extracted and used as gold standards for evaluation. According to the annotation guideline, we only try to predict GO terms based on the text from the abstract, results section, discussion section, and conclusion section.

We use the standard measures of precision ($\frac{TP}{TP+ FP}$), recall ($\frac{TP}{TP+ FN}$) and F1-score ($2\ast \frac{precision\ast recall}{precision+ recall}$) for evaluations, where TP stands for true positive (PMID, Gene ID and GOID triplet that is correctly predicted by the method), FP stands for false positive (PMID, Gene ID and GOID triplet that is incorrectly predicted by the method) and FN stands for false negative (PMID, Gene ID and GOID triplet that should be predicted but is missed by the method).

To best of our knowledge, previous approaches for GO annotation all use a single approach for annotations of all types of GO terms, and there is no system for automatic GO annotation publicly available. Thus, there is no other system that is appropriate for comparison. Table 2 shows the precision, recall and F1-score using our approach to predict GO terms from CC Domain for given genes. Our approach obtains a F1-score of 71%, a precision of 91% and a recall of 58%.

**Table 2:** Performances of predicting GO terms for given genes

Precision	Recall	F1-score
91%	58%	71%

## Discussion

We analyzed the false positives (FPs) and the false negatives (FNs) and found the FPs were mainly due to the errors of dependency parsing and entity recognitions. Since many of the annotations used text evidence from multiple sentences, while our approach mainly captures the ones from one single sentence, majority of the FNs were due to fact that we didn’t anaphoric expressions such as “both proteins” and “it”. For example, in sentence “We monitored the subcellular localization of ZmOST1-GFP and ZmSNAC1-GFP constructs in Nicotiana benthamiana and found that both proteins are localized in the nucleus and the cytoplasm of tobacco epidermal cells.” from PMID 23469147 (PMC3585266). If we can anaphoric “both proteins” to “ZmOST1” and “ZmSNAC1”, then we can capture the relations for the two proteins to location “nucleus” and “cytoplasm”, based on the syntactic dependencies. The remaining FNs correspond to cases where there is no clear-cut syntactic dependency between the protein and other entities. Thus, the syntactic dependency driven approach is unlikely to have captured these cases. We found sentential co-occurrence with careful restrictions might help to improve the recall without hurting the precision. This can be a potential future investigation topic.

Evaluation results show treating GO annotation as relation extraction tasks can yield good performance. This idea can also be applied to other types of GO terms. For example, based on our study, we believe our approach can be especially useful for two major sub-hierarchies of the Molecular Function domain: protein binding terms and catalytic activity terms. Annotation with terms from these two sub-hierarchies can be benefit from: (1) extracting cases where a protein binds to other proteins, and (2) extracting cases where a protein is involved in catalytic activity. We believe approaches following these processes can yield high precision as well, thus can assist in the process of GO annotation.

## Conclusions

In this paper, we have described a novel approach to automatically annotate genes using GO terms from cellular component domain. In contrast to previous approaches, we treat annotation with different types of GO terms as individual subtasks, where we cast each subtask as relation extraction between gene and other entities: (1) extract cases where a protein is in a subcellular location, and (2) extract cases where a protein is a subunit. Evaluation results shows that our approach achieves a F1-score of 71% (91% precision) in predicting GO terms for given genes. Thereby it can be used to accelerate the process of GO annotation for the bio-annotators. In the future, we plan to investigate how to improve the recall of our approach. One possible direction is to apply sentential co-occurrence heuristic with careful restrictions.

## Abbreviations

BP: Biological Process
CC: Cellular Component
FN: False Negative
FP: False Positive
GO: Gene Ontology
MF: Molecular Function
MOD: Model Organism Database
